# Gastrointestinal Malignancy Presenting with a Virchow's Node in a Patient with Rothmund-Thomson Syndrome

**DOI:** 10.1155/2018/7536832

**Published:** 2018-10-25

**Authors:** Kara Nadeau, Michele Brule

**Affiliations:** ^1^Faculty of Medicine, University of Ottawa, Ottawa, Ontario, Canada; ^2^Department of General Surgery, Northern Ontario School of Medicine, Sudbury, Ontario, Canada

## Abstract

Rothmund-Thomson syndrome is a genetic disorder with characteristic findings in childhood as well as a predisposition to osteosarcoma, skin cancer, and hematological malignancy. We present the first reported case of duodenal malignancy in a patient with Rothmund-Thompson syndrome. An enlarged Virchow's node was noted and an advanced duodenal adenocarcinoma was diagnosed shortly thereafter. The features of Rothmund-Thomson syndrome are discussed, as well as current management and screening guidelines for duodenal adenocarcinoma.

## 1. Case Report

A 28-year-old male patient with Type II Rothmund-Thomson syndrome presented to his physician with an enlarged left supraclavicular (Virchow's) node. Due to his syndrome, he had a small build, areas of skin hyperpigmentation, early skin aging with actinic keratoses, sparse thin hair, abnormal skeletal development, and osteoporotic brittle bones with a history of 18 fractures. Previously, at age 25, a lesion at the base of the penis was excised which was diagnosed as Bowen's disease. At the time of presentation, a neck ultrasound confirmed an enlarged 1.8 cm left supraclavicular node which appeared to be avascular. A chest X-ray and abdominal ultrasound were performed at that time which were normal. An outpatient referral was made to a local surgeon.

Two months later, he presented to a community hospital with a three-week history of progressively worsening postprandial vomiting and upper abdominal pain. At that time, he was not tolerating any oral intake. A repeat ultrasound demonstrated a concerning mass on the left lateral aspect of the aorta. An esophagogastroduodenoscopy was done urgently, and a polyp was found in the 2nd part of the duodenum as well as an inflamed and friable obstructing lesion in the 3rd part of the duodenum. The scope could not be advanced beyond this point. Biopsies taken at the time of endoscopy revealed high grade invasive mucinous adenocarcinoma of the signet ring cell type with neuroendocrine features at the site of the obstructing lesion. The biopsies of the polyp demonstrated high grade intramucosal mucinous adenocarcinoma arising within a tubulovillous adenomatous polyp.

He was transferred to our institution to undergo further imaging and management. At that time, his functional status was poor. He had lost approximately 25 lbs and had an ECoG performance status of 3 to 4. On examination, a fullness in the epigastrium was noted. The CT scan demonstrated a large distal duodenal mass with extensive lymphadenopathy, as well as a nodule in the right upper lobe of the lung. Attempts to place a duodenal stent across the lesion were unsuccessful. He underwent palliative radiotherapy and subsequently underwent a laparotomy and gastrojejunostomy to bypass the obstructing lesion. The patient declined systemic chemotherapy and was treated with palliative intent, as per his goals of care, which allowed him to return home to be with his family.

He developed Herpes Zoster shortly after his discharge and was treated as an outpatient with oral antiviral therapy. Two months after his diagnosis of duodenal adenocarcinoma, he presented to the hospital with significant shortness of breath. Additional imaging was done which revealed multiple masses in the right lung, significantly increased mediastinal adenopathy compressing the pulmonary artery, postobstructive pneumonitis in the right lower lobe, and airspace disease suspicious for aspiration bilaterally. He progressively worsened despite supportive care and passed away shortly thereafter.

## 2. Discussion

Rothmund-Thomson syndrome (RTS) is a rare disorder that presents in childhood with characteristic skin findings, short stature, and congenital skeletal malformations [1]. It was described by both German ophthalmologist Rothmund (1898) as well as British dermatologist Thomson (1936), and approximately 300 cases have been described in the published literature to date [1, 2]. A subset of RTS patients also have a predisposition to sarcomas, skin cancers, and hematological malignancies [1, 3]. A single case of gastric carcinoma has been reported in the literature, dating back to 1975, which is the only other reported case of gastrointestinal malignancy in RTS [4]. Thus, this is the first reported case of duodenal adenocarcinoma in a patient with RTS.

Stinco et al. identified 61 patients with RTS and malignancy and found the mean age at diagnosis of malignancy to be 18.7 years, with ages ranging from 3 to 49 years [5]. The most frequently identified malignancy was osteosarcoma, especially in the lower limbs, at a mean age of 14 years [5]. Epithelial tumors such as squamous and basal cell carcinomas presented most often in adulthood with a mean age of 34 years [5]. Gastrointestinal abnormalities have been described in patients with RTS, including esophageal stenosis, pyloric stenosis, annular pancreas, anal atresia, and rectovaginal fistula [6].

RTS has been subdivided into two subgroups. RTS type II has been recognized as a genetically transmitted disorder of the* RECQL4* gene, affecting the RECQL4 DNA helicase and thus leading to genomic instability [1]. Affected individuals characteristically have facial erythema, poikiloderma (altered skin pigmentation, atrophy, and telangiectasias), hyperkeratosis, small stature, and skeletal defects [3]. RTS type I is defined by the absence of* RECQL4* mutations in the presence of the RTS phenotype; the genetic defect of these individuals is currently unknown. This subgroup of patients also differs from RTS type II as they do not appear to have an increased risk of malignancy [1]. However, patients with type I have an increased risk of bilateral juvenile cataracts [1]. Patients with RTS are routinely managed by an interdisciplinary team and screened for skeletal abnormalities, cataracts, osteosarcoma, and skin cancer in childhood [3].

Neoplastic adenopathy at Virchow's node is a well-known indicator of abdominal malignancy [7]. However, duodenal adenocarcinoma is a rare cause and accounts for less than 0.5% of gastrointestinal malignancies [7, 8]. Duodenal adenocarcinoma may also present with epigastric pain, gastric outlet obstruction, jaundice, cholangitis, melena, anemia, abdominal mass, and weight loss, or it may be incidental finding in asymptomatic patients [8, 9]. It presents at a median age of 64 years [8]. While curative resection has been shown to improve survival rates, most cases are diagnosed at an advanced stage. In a retrospective analysis of duodenal carcinoma by Lee et al., median survival rates were reported to be approximately 6 months in patients who were not candidates for curative surgery and approximately 25 months in patients who underwent curative surgery [8].

Treatment of duodenal adenocarcinoma depends on the extent of disease at presentation. Curative procedures for duodenal tumors include pancreaticoduodenectomy and segmental resection [8, 9]. Factors associated with poor overall survival in patients who undergo curative resection include the presence of lymphovascular invasion, positive nodal status, and lack of R0 resection [8, 9]. In patients with unresectable metastatic disease, surgical intervention may be indicated for palliation of obstruction or refractory bleeding [9]. The use of adjuvant chemotherapy and radiation therapy has not been shown to increase survival in patients with duodenal adenocarcinoma, although data is limited and there are no clear recommendations [10, 11]. Patients with advanced disease at presentation may benefit from palliative chemotherapy, with increased survival from 2 to 12 months [10].

Multiple genetic syndromes affecting genomic stability have been identified as risk factors for duodenal cancers, including familial adenomatous polyposis (FAP), Lynch syndrome (also known as hereditary nonpolyposis colorectal cancer), and Peutz-Jeghers syndrome [10]. Screening methods using duodenoscopy and endoscopic ultrasound have been proposed for patients with these risk factors, though they have not demonstrated any benefit in survival, and therefore recommendations for routine surveillance vary [12, 13]. In RTS, screening would not be recommended given the rarity of gastrointestinal malignancy in this population.

In conclusion, this is the first reported case of duodenal carcinoma in a patient with RTS. Duodenal malignancy is relatively uncommon, and the prognosis is poor, especially with advanced disease at diagnosis. Increased awareness of the risk of gastrointestinal malignancy in patients with rare genetic alterations such as RTS is important to improve detection and outcomes in these patient groups.
